# Supplementary material for: Examining interviewer bias in medical school admissions: The interplay between applicant and interviewer gender and its effects on interview outcomes
Source: PLoS One. 2024 Aug 26;19(8):e0309293. doi: 10.1371/journal.pone.0309293 (PMC11346660; doi:10.1371/journal.pone.0309293)
Supplement: S1 File — (PDF) [file pone.0309293.s001.pdf]

Examining interviewer bias in medical school admissions:
The interplay between applicant and interviewer gender and its effects on interview outcomes

---

Online Attachment

Additional information on the regression models

OLS regressions for interview grades

In equation (1), we estimate the interview grade (in the range of 0-15) for subject i interviewed by selection committee type $j \in [0,1,2]$ (0 := MM, 1 := MF, 2 := FF) in year $t \in$ [2006, 2007, ..., 2019], conditional on explanatory variables that may influence the outcome. Our main predictors of interest include subjects' gender (a binary indicator if the interviewee was female or not) and the commission type j. The interaction between gender and commission type (β_3) is of particular importance, as it gives the predicted and adjusted interview score for CF (CM) conditional on whether she or he was interviewed by MM, MF, or FF.

$$\widehat{interview}\,grade_{ijt} = \beta_0 + \beta_1 commission_j + \beta_2 female_i + \beta_3 commission_j \times female_i + \beta_4 abitur_i + \beta_k \sum_{k=6}^{l} controls_i + FE_t + u_i \quad (1)$$

Since the ranking that determined whether an applicant was selected or not was formed based on the interview and the Abitur grade, we include the latter in the regression model. Other control variables include the waiting semester and a binary indicator for voluntary service. We further include interview year fixed effects (FE_t) to control for time-dependent trends or year-specific circumstances, such as increases in Abitur grades over time or commission type distribution.

Table 3 in the main text is based on equation (1) with the variables described above. In Table A1 below, we extend the model by including age and school type as additional covariates. This reduces the number of observations.

Figure 2 in the main text, i.e. the illustration of β_3 in equation (1) with the calculation of partial effects, is constructed by taking the partial derivative of the outcome variable with respect to commission type j. The outcome variable itself is adjusted for the covariates and the Abitur grade included in eq. (1)

Figure 3 (again the illustration of partial derivatives) in the main text is derived by extending eq. (1): Interrelating the indicator 'voluntary service' multiplicatively with the gender-commission type interaction yields regression equation (2):

$$\widehat{interview}\, grade_{ijt} = \beta_0 + \beta_1 commission_j + \beta_2 female_i + \beta_3 voluntary\ service_i + \beta_4 commission_j \times female_i + \beta_5 commission_j \times voluntary\ service_i + \beta_6 female_i \times voluntary\ servuce_i + \beta_7 commission_j \times female_i \times voluntary\ service_i + \beta_8 abitur_i + \beta_k \sum_{k=9}^{l} controls_i + FE_t + u_i \quad (2)$$

Probit regressions for selection probabilities

Figure 4 in the main text is derived analogous to Figure 2. However, the continuous variable 'interview grade' is replaced with the binary indicator whether a study place was offered or not. Now, instead of estimating an OLS model, a non-linear probit model is employed. Thus, as a variation of eq. (1), we estimate the probability (*Pr*) of being selected (1 = yes, 0 = no) for subject *i*, conditional on the same predictors and the same vector ***X*** of explanatory variables that may influence the outcome, here *Pr* ($selection_{ijt} = 1 \mid \mathbf{X} = x$), as in eq. (1).
A full regression output is presented in Table A2 (probit coefficients) and Table A3 (partial effects).

Figure 5 in the main text illustrates the predictive probability of a successful interview at each Abitur grade. This is achieved by calculating partial effects of $selection_{ijt} = 1$ with respect to commission type j at the observed Abitur grades using equation (3):

$$\widehat{selection}_{ijt} = \beta_0 + \beta_1 commission_j + \beta_2 female_i + \beta_3 abitur_i + \beta_4 commission_j \times female_i + \beta_5 commission_j \times interview_i + \beta_6 female_i \times interview_i + \beta_7 commission_j \times female_i \times abitur_i + \beta_8 interview_i + \beta_k \sum_{k=9}^{l} controls_i + FE_t + u_i \quad (3)$$

Results for regressions using eq. (3) are presented in columns (3-4) in Tables A2 and A3.

Table A1: Regression analysis for interview scores with additional covariates

	(1) Interview grade	(2) Q-Pers	(3) Q-Pro	(4) Q-Act	(5) Q-Ovr
Selection committee type (reference is MM)					
MF	-0.305	-0.167	-0.188	-0.051	-0.155
	(0.200)	(0.225)	(0.234)	(0.227)	(0.229)
FF	0.630	0.875	0.701	0.146	0.406
	(0.687)	(0.695)	(0.692)	(0.805)	(0.744)
Gender of applicant (reference is MM)					
CF	-0.210	-0.079	-0.282	-0.263	-0.203
	(0.139)	(0.159)	(0.166)	(0.162)	(0.163)
Interaction terms (reference is MM × CM)					
MF × CF	0.171	0.218	0.377	0.222	0.267
	(0.233)	(0.260)	(0.271)	(0.263)	(0.265)
FF × CF	-0.718	-1.020	-0.722	-0.068	-0.589
	(0.789)	(0.795)	(0.848)	(0.906)	(0.860)
Age	-0.089*	0.009	-0.020	-0.048	-0.017
	(0.032)	(0.037)	(0.040)	(0.038)	(0.038)
Gymnasium	0.467*	0.472*	0.411*	0.526*	0.467*
	(0.162)	(0.179)	(0.188)	(0.182)	(0.182)
Abitur grade	0.187*	0.186*	0.154	0.167*	0.170*
	(0.069)	(0.081)	(0.086)	(0.084)	(0.084)
Voluntary service	0.908*	1.023*	1.161*	1.099*	1.147*
	(0.152)	(0.165)	(0.171)	(0.168)	(0.169)
Waiting time	0.031	-0.065	-0.009	-0.022	-0.037
	(0.039)	(0.042)	(0.043)	(0.043)	(0.044)
Year FE	YES	YES	YES	YES	YES
Observations	3970	3305	3305	3305	3305
R^2	0.026	0.024	0.027	0.026	0.025

Notes: OLS regressions for the overall interview grade and the separate conversation topics. Robust standard errors are in parentheses. Year-fixed effects (FE) are jointly significant in all specifications (Wald-Test). See Table 3 in the main text for regression models excluding Age and School type. The underlying regression model is described in equation (1) of the Online Attachment. CM: candidate is male; CF: candidate is female; MM: all-male selection committee; MF: mixed-gender selection committee; FF: all-female committee. *: $p<0.05$

Table A2: Probit regressions for Selection = 1, coefficients are reported

Dep. Var.: study place (yes/no)	(1)	(2)	(3)	(4)
**Reference category: MM**				
MF	0.376	-0.115	-2.629	2.636
	(0.263)	(0.539)	(2.992)	(5.065)
FF	2.785*	3.367*	47.865*	119.537*
	(0.561)	(0.649)	(9.714)	(28.617)
**Reference category: CM**				
CF	0.367	0.529	0.965	3.746
	(0.215)	(0.369)	(2.284)	(3.134)
Abitur grade	7.652*	18.938*	7.650*	20.070*
	(1.275)	(2.968)	(1.251)	(3.114)
Interview grade	7.239*	17.988*	7.319*	18.990*
	(1.170)	(2.817)	(1.158)	(2.961)
**Interaction terms (Ref.: MM, CM)**				
MF × CF	-0.127	0.428	-1.393	-11.776*
	(0.323)	(0.617)	(3.615)	(5.794)
FF × CF	-3.247*	-4.247*	-46.689*	-59.470*
	(0.760)	(1.061)	(9.858)	(22.630)
MF × Abitur grade			0.243	-0.228
			(0.244)	(0.436)
FF × Abitur grade			-3.535*	-9.608*
			(0.744)	(2.257)
CF × Abitur grade			-0.046	-0.255
			(0.184)	(0.254)
MF × CF × Abitur grade			0.091	0.966*
			(0.290)	(0.488)
FF × CF × Abitur grade			3.415*	5.311*
			(0.755)	(1.781)
Voluntary service	0.001	0.510	-0.035	0.539
	(0.249)	(0.365)	(0.245)	(0.341)
Waiting time	0.147	0.141	0.146	0.149
	(0.078)	(0.111)	(0.077)	(0.130)
Age at interview		-0.017		-0.039
		(0.161)		(0.196)
Abitur at Gymnasium		-0.131		-0.049
		(0.348)		(0.383)
Year FE	YES	YES	YES	YES
Observations	5036	3970	5036	3970
Pseudo R^2	0.955	0.975	0.956	0.977

Notes: Coefficients from probit regressions with the dependent variable Selection = 1 are reported in this table. Robust standard errors are in parentheses. Year fixed effects (FE) are included and jointly statistically significant in all specifications (Wald-Test). Models (2) and (4) include participants' age and educational background as additional control variables. Data were not available for the years 2006, 2007 and 2019. Thus, the number of observations is smaller. Figure 4 in the main text is based on model (1), Figure 5 on specification (3). MM: all-male selection committee; MF: mixed-gender committee; FF: all-female selection committee; CM: male candidate; CF: female candidate. *: $p<0.05$

Table A3: Probit regressions for Selection = 1
marginal effects are reported

	(1)	(2)	(3)	(4)
**Reference category: MM**				
MF	0.005	0.002	0.005	0.002
	(0.003)	(0.002)	(0.003)	(0.002)
FF	0.009	0.002	0.013	0.023*
	(0.007)	(0.006)	(0.007)	(0.003)
**Reference category: CM**				
CF	0.005	0.007*	0.004	0.008*
	(0.003)	(0.003)	(0.003)	(0.004)
Abitur grade	0.142*	0.184*	0.142*	0.182*
	(0.004)	(0.014)	(0.004)	(0.014)
Interview grade	0.134*	0.175*	0.134*	0.172*
	(0.003)	(0.014)	(0.003)	(0.013)
Voluntary service	0.000	0.005	-0.001	0.005
	(0.005)	(0.003)	(0.005)	(0.003)
Waiting time	0.003	0.001	0.003	0.001
	(0.002)	(0.001)	(0.002)	(0.001)
Age at interview		-0.000		-0.000
		(0.002)		(0.002)
Abitur at Gymnasium		-0.001		-0.000
		(0.003)		(0.003)
Year FE	YES	YES	YES	YES
Observations	5036	3970	5036	3970

Notes: The numbers reported in this table are partial derivatives of the dependent variable with respect to all parameters and interactions reported in Table A2. These marginal effects can be interpreted as the response of the dependent variable in percentage-points to a binary explanatory variable being = 1, or the response of the dependent variable in percent for continuous explanatory variables. Robust standard errors are in parentheses. Further table notes from Table A2 apply here as well. *: $p<0.05$
