# Supplementary material for: Examining interviewer bias in medical school admissions: The interplay between applicant and interviewer gender and its effects on interview outcomes
Source: PLoS One. 2024 Aug 26;19(8):e0309293. doi: 10.1371/journal.pone.0309293 (PMC11346660; doi:10.1371/journal.pone.0309293)
Supplement: Attachment — Submitted filename: Response to Reviewers.docx [file pone.0309293.s003.docx]

Examining interviewer bias in medical school admissions:

The interplay between applicant and interviewer gender and its effects on interview outcomes

**Detailed responses to comments and suggestions**

**by the academic editor and two reviewers**

Original, unedited comments by the academic editor and two reviewers are indicated in blue script. Our detailed responses to all comments and suggestions are indicated in red script.

We thank both reviewers for the time invested in reviewing the manuscript and their many helpful suggestions, which helped improving our work!

**Academic Editor**

***When submitting your revision, we need you to address these additional requirements.***

1. Please ensure that your manuscript meets PLOS ONE's style requirements, including those for file naming. The PLOS ONE style templates can be found at

https://journals.plos.org/plosone/s/file?id=wjVg/PLOSOne_formatting_sample_main_body.pdf and

<https://journals.plos.org/plosone/s/file?id=ba62/PLOSOne_formatting_sample_title_authors_affiliations.pdf>

We double-checked the manuscript and confirm that it meets the style requirements of PLOS ONE. Minor changes have been applied to table and figure captions (e.g. “Fig” instead of “Figure”).

2. For studies reporting research involving human participants, PLOS ONE requires authors to confirm that this specific study was reviewed and approved by an institutional review board (ethics committee) before the study began. Please provide the specific name of the ethics committee/IRB that approved your study, or explain why you did not seek approval in this case.

Once you have amended this/these statement(s) in the Methods section of the manuscript, please add the same text to the “Ethics Statement” field of the submission form (via “Edit Submission”).

For additional information about PLOS ONE ethical requirements for human subjects research, please refer to <http://journals.plos.org/plosone/s/submission-guidelines#loc-human-subjects-research>.

In the “Data” subsection of the “Materials and methods” section of the manuscript, we gave a detailed description why our study was exempt from separate approval by an ethics committee. We analyzed only retrospective, administrative and fully anonymized data that does not include human participants in a clinical sense. The use of such data for research and quality assurance purposes is governed by MHH study regulations (that passed all governing bodies at MHH) and higher education laws detailed in the manuscript, the “Ethics statement” field of the submission form and the “Human Subjects Research Checklist” we attached to the submission.

To make our explanations more prominent in the manuscript, we have put our statement in a new, separate subsection in the “Materials and methods” section, named “Ethics statement”.

3. Please provide additional details regarding participant consent. In the ethics statement in the Methods and online submission information, please ensure that you have specified what type you obtained (for instance, written or verbal, and if verbal, how it was documented and witnessed). If your study included minors, state whether you obtained consent from parents or guardians. If the need for consent was waived by the ethics committee, please include this information.

See comment 2: Participant consent is not applicable as we analyzed retrospective, administrative data. We provided details regarding participant consent in the manuscript, the “Ethics statement” of the submission form and the “Human Subjects Research Checklist”.

The points raised in comments 2 and 3 were checked in and passed the editorial review phase back in December 2023.

4. In the online submission form, you indicated that [Data and code can be made available by the corresponding author upon reasonable request].

All PLOS journals now require all data underlying the findings described in their manuscript to be freely available to other researchers, either 1. In a public repository, 2. Within the manuscript itself, or 3. Uploaded as supplementary information.

This policy applies to all data except where public deposition would breach compliance with the protocol approved by your research ethics board. If your data cannot be made publicly available for ethical or legal reasons (e.g., public availability would compromise patient privacy), please explain your reasons on resubmission and your exemption request will be escalated for approval.

We are able to provide the dataset in Excel-format as supplementary information.

5. Please review your reference list to ensure that it is complete and correct. If you have cited papers that have been retracted, please include the rationale for doing so in the manuscript text, or remove these references and replace them with relevant current references. Any changes to the reference list should be mentioned in the rebuttal letter that accompanies your revised manuscript. If you need to cite a retracted article, indicate the article’s retracted status in the References list and also include a citation and full reference for the retraction notice.

The reference list is complete and correct.

**Reviewer #1**

Thanks for the interesting data about a relevant topic. The reader need some important informations in the methods section and also the discussion would benefit from some more thoughs.
Language
I am not a native speaker myself, but I had the impression, that the language could be improved. E.g. “Motivated by the described shortcomings of personal interviews…,” or: Or (page 15, line 4): ….”Missing for some years are data on ratings in the….” sounds very “German”. Same with “..One often-used instrument is the Multiple Mini-Interview….” Better: a frequently used instrument as the MMI….”. Please check the language once more through the whole paper.
Please correct also upper and lower case letters (e.g. in methods: “Results chapter”)

In the process of revising the manuscript, we made several smaller changes to wording, formulations and typos. This includes the sentences the reviewer referred to in the comment.

**Abstract**

Ok, if no subheadings are needed

According to the PLOS ONE Style Guide, the Abstract should not use subheadings.

**Introduction**

Page 10, 3 paragraph: delete the first word “in” or “has been found that….”

We have implemented this suggestion.

Same paragraph: what sort of “test” is meant here: “……in favor of the latter males scored higher in the test” ? interviews? MMI? Cognitive or psychometric tests?

“Test” refers to the admission test mentioned earlier in the same sentence. We give more context in the revised manuscript.

Same paragraph: “For the admission process at Brown University, [28] showed that…” please add authors name or “it was shown, that….”.

We have implemented this suggestion.

**Research objectives:**

delete the sentence: “(as discussed in the previous section)”

Okay.

Some sentences can moved into the discussion
e.g.: into “strength”: data from 15 years,
or into “limitations”: one-site study, no detailed information about rating

We implemented this suggestion by organizing the “Discussion” chapter into subsections. Some statements of the research objectives were moved to the new subheading “Limitations” within the “Discussion” chapter. Many of the “strengths” we describe are not positive findings from our analysis, but motivated our approach and were integral to the setup of our study, e.g. the broader database (in contrast to related studies) and the analysis of interaction effects. Therefore, we decided to leave these aspects in the introduction, because they describe and motivate our research objectives.

**Methods**

In the methods-section, several questions are not answered so far:
Did the interviewers know the candidate's A-level grades beforehand? Or other information about the candidates?

Interviewers received a type of CV (based on information provided by the candidates) which included details such as education, school subjects, extracurricular activities, vocational training, and experiences as exchange students. However, we do not have access to these questionnaires. The high school leaving grade was not asked on the form. Whether the interviewers had knowledge of the high school leaving grade or inquired about it during the interview is unknown to us. As we discuss at the end of the paper, the results suggest that this was likely the case. Overall, the high school leaving grade and the interview score are uncorrelated. We have added a brief description of the questionnaire with the information available to the interviewers to the manuscript.

What was the training like when it took place?

The brief answer is that we do not know, as there are no protocols. In the manuscript, we have mentioned that interviewer trainings were not standardized. Anecdotes from a few involved persons suggest that these trainings had a very informative character and evolved around proceedings and an explanation of the different parts of the interviews. There were, e.g., no simulation of interviews.

Which professional groups did the interviewers come from?

As described at the top of page 8 (manuscript numbering), each committee consisted of two physicians who were also faculty members at Hannover Medical School (MHH). In the early years, the interviews were conducted exclusively by professors. Later on, habilitated faculty and other teaching staff (e.g., senior physicians) were also allowed to conduct interviews.

Care was taken to ensure that the committee members belonged to different sections of the MHH (large and small clinical subjects, theoretical subjects), with at least one member being clinically active. We have added this information to the manuscript.

How many minutes did each interview last?

We thank the reviewer for pointing to this missing information. Interviews lasted at least 20 and no longer than 30 minutes. We have added this to the manuscript.

What is the maximum number of consecutive interviews per day/per session? Please discuss later possible exhaustion after a long periods as it is known from OSCES or exams.

This question is indeed very interesting and essentially represents a separate research question, which would go beyond the scope of the present manuscript if added. At the end of the discussion section (now subheading “Contextualization”), we reference papers that deal with “interviewer fatigue”.

Based on available information, we estimate that interviewer pairs conducted a maximum of eight interviews per day in the early years and were typically assigned for no more than three days (and not on consecutive days). As more interviewers and a higher number of commissions were added in later years, the workload tended to decrease.

Were all candidates binary or were some divers?

All candidates were either male or female.

Why data collection ended in 2019?

Student selection via interviews at MHH ended after 2019 (we added this information in the subsection “Data”). This was mainly due to a landmark ruling by the German federal Supreme Court, which demanded a general overhaul of student selection processes in Germany. In a transition period, places at medical school were only centrally allocated. After the transition period, the MHH decided not to return to the interview format.

Were the intervievers informed about the candidates success later?

Not to our knowledge. The creation of the ranking lists, notification of candidates etc. likely took some time.

**Results**

good

No revision required.

**Discussion**

Please add subheadings.

We thank Reviewer #1 for this suggestion. We divided the “Discussion” chapter into subsections.

P 20., last line: something missing? The reader expects a quotation („Goldberg claims…“).

This refers to the previous sentence (Goldberg [38]). To avoid confusion, we have added the reference at that point as well.

How is he distribution between male and female students at MHH in the years of study? Perhaps female interviewers had a sort of “hidden agenda” to increase the proportion of male students? Please add some ideas about further research

A very interesting and valid thought. In all the years analyzed in our paper and even before, approximately two-thirds of the students were women. This is the case nationwide in Germany and roughly corresponds to the distribution of the interviewees. Even if the interviewers did not know the exact number, they would be well aware that many more women attend lectures, seminars, etc., than men. In the discussion, we address the fact that more women study medicine because they achieve better school grades than men and therefore have better chances of securing a place in all selection processes that consider school leaving grades. In the discussion, we argued that all-female committees have perhaps explicitly or implicitly wanted to compensate for this disadvantage of men who performed very well in the interview. This could also be applied to the gender distribution, but it cannot be proven empirically (at least not with our data).

No conclusion

We decided to add a short concluding statement (or take-home message, see Reviewer #2) at the end of the manuscript (separate subheading in the “Discussion” chapter).

**Literature**

Ok

No revision required.

**Graphs/tables**

good

No revision required.

**Reviewer #2**

In this study “Examining interviewer bias in medical school admissions: The interplay between applicant and interviewer gender and its effects on interview outcomes” , the authors investigate gender-related interviewer bias in medical school admission interviews using data from 5,200 individuals who participated in selection interviews between 2006 and 2019.
I appreciate the time and effort spent by the authors on this important contribution to the literature. It is a study that will shed light on future research in this field. However, I suggest some changes before publication. These are minor technical issues that have come to my attention. The suggestions are listed below.

1. On Page 2, there is no need for "see reviews by" in the last sentence. Sources [1-4] are already mentioned. Please delete this statement.

We agree with this suggestion and have removed “see reviews by”.

2. In the third sentence on Page 3, there is no need for "reviews by" and "See also". Instead, list the sources side by side.

Okay, see above.

3. All of the following sources are written separately from each other. Please write them
[10] [2],
[14] [17-18]
[31-32] [16],
[44-45] [21].

We do not quite understand what Reviewer #2 is suggesting here. However, we made minor changes, e.g. writing [2, 10] instead of [2] [10].

4. On page 4, p. 1440, is this necessary? it is already referenced. This style of referencing is not customary for articles, nor is it necessary.

The mentioned passage is a direct quote, and we consider it appropriate and correct to provide the page number for this direct quote.

5. “For a start, we briefly present previous evidence on gender biases in (selection) interviews for study programs and residency trainings.”
I do not think these expressions are necessary, they even disrupt the flow. Going directly to the subject to be explained will help to understand more clearly.

We agree with the reviewer’s suggestion and have removed this sentence from the manuscript.

6. It is sufficient to mention only the purpose of the research in the "Research objectives" section on page 5. Including the following section in the strengths and limitations section of the research will increase fluency. It should be moved to the relevant section
“Similar to the few existing studies addressing biases in university admissions interviews (as discussed in the previous section), our investigation is a one-site study. Consequently, the results may not be generalizable due to the specificity of the selection procedure. However, our study introduces several innovations and expansions compared to pre- vious research:
• Our data encompasses not only individual years but the analysis of all interviews con- ducted over a span of 15 years, providing a significantly larger dataset and greater rep- resentativeness of the examined selection process.
• The focus of the paper is on interaction effects between the gender of interviewees and interviewers and goes beyond simple group-comparisons
• Instead of merely reporting differences, we aim to provide explanatory approaches. For Instead of merely reporting differences, we aim to provide explanatory approaches. For instance, we examine whether certain characteristics, such as the presence of voluntary service, can account for differences and whether these are gender-specific.”

See also point 7 below: We agree with the reviewer’s suggestion insofar that the limitations we describe in the “Introduction” have been moved to a separate subheading in the “Discussion” chapter. However, the broader database, analysis of interaction effects etc. (which can be understood as “strengths”) are integral to our research design and describe how we want to advance the literature on interview bias with our study. Thus, we decided to leave these points in the “Research objectives” section.

7. Likewise, the first paragraph on page 6 should be moved to the limitations section
“Some constraints of our study should be acknowledged upfront. Our study does not aim to draw clear conclusions about whether any differences represent a "true" bias based on favoritism or discrimination e.g. due to gender-specific stereotypes. This is hindered by the lack of infor- mation and documentation regarding the reasons behind given evaluations.
We do also not contribute to biases, specifically gender bias, on a conceptual or theoretical level. The focus is on a positive, empirical examination of potential interviewer biases embed- ded in the selection of applicants into medical school.”

Please see our answer to point 6.

8. Write MHH open under Figure 1
(Figure 1: The student selection procedure at MHH)

We are not sure what is meant with “open”. We have changed the title of Fig 1 to “The student selection procedure at Hannover Medical School (MHH).”

9. Page 21 --- A study by [37] ------ work done by whom? (please specify author)

Agreed, adjusted.

10. Include the strengths and limitations of the study after the discussion section.

Please see our answer to point 6 raised by reviewer #2. We moved the limitations to a separate subsection in the “Discussion” chapter.

11. Finally, what is your take-home message? Please state it more clearly.

We decided to add a short concluding statement (see also Reviewer #1) at the end of the manuscript.

Good work

We appreciate the positive assessment, thank you!
